# Leg Spin or Off Spin? Orthodox or Unorthodox?—An In‐Depth Examination of Bowling–Batting Match‐Ups and the Effectiveness of Spin Variations in International and Franchise T20 Cricket

**DOI:** 10.1002/ejsc.12296

**Published:** 2025-04-10

**Authors:** Samuel Kerruish, Allistair McRobert, Mikael Jamil

**Affiliations:** ^1^ School of Allied Health Sciences University of Suffolk Ipswich UK; ^2^ School of Sport and Exercise Sciences Liverpool John Moores University Liverpool UK

**Keywords:** googly, key performance indicators, match analysis, performance analysis, spin bowling

## Abstract

In this study, 23,084 balls bowled by elite level spin bowlers across six competitions were analysed in order to fulfil two main aims; (1) investigate whether the type of spin bowler presents any tactical advantages and (2) investigate the most effective type of spin bowling variation. The results of logistic regression analyses revealed significant relationships between specific bowler–batter match‐ups and runs conceded (*p* < 0.001). Specifically, opposing match‐ups where the ball naturally spins away from right‐handed and left‐handed batters were revealed to be a particularly effective strategy at restricting runs conceded. Right‐handed leg‐spin bowlers are revealed to be significantly more likely to take the wickets of right‐handed batters. Results also revealed that the ‘googly’ and ‘carrom ball’ variations are particularly effective at both, restricting runs scored and taking wickets when they are bowled to right‐handed batters (both *p* < 0.001). Evidence suggests that certain bowler–batter match‐ups present some tactical advantages and should therefore be taken into consideration in T20 cricket. Furthermore, the results also highlight the value of a wrist spinner capable of bowling ‘googly’ or ‘carrom ball’ variations. The findings of this study could potentially influence team selection, strategies, recruitment policy and general coaching practice.

**Summary**

- Opposing match‐ups where the ball naturally spins away from both right‐handed and left‐handed batters is a particularly effective strategy towards restricting the number of runs conceded.
- Right‐handed leg‐spin bowlers are revealed in this study to be significantly more likely to take the wickets of right‐handed batters.
- The ‘googly’ and ‘carrom ball’ variations are particularly effective at both, restricting runs scored and taking wickets when they are bowled to right‐handed batters (both *p* < 0.001), highlighting the value of a specialist wrist spinner capable of bowling these variations in T20 cricket.
- Certain bowler–batter match‐ups do present some tactical advantages and should therefore be taken into consideration in T20 cricket match strategy.

## 1 Introduction

Key performance indicators (KPI's) need to be successfully and regularly achieved in order to obtain positive outcomes for a team or athlete (Hughes and Bartlett 2002). In terms of cricket research, several previous studies have identified the key performance indicators that greatly influence batting success, chief of which, includes the batter's ability to score runs and frequently clear the boundary, especially in limited over cricket (Douglas and Tam 2010; Irvine and Kennedy 2017; Jamil et al. 2022; Petersen et al. 2008). Therefore, the objective of bowlers is to restrict the number of runs conceded whilst also attempting to take wickets of their opposing batters (Douglas and Tam 2010; Jamil et al. 2021; Mehta et al. 2022). Although the bulk of previous research into bowling has focused on fast bowlers (Vickery et al. 2017), spin bowling has attracted some research, particularly from a biomechanical perspective (Beach et al. 2016; Chin et al. 2009). When comparing offspin and leg spin, Beach et al. (2016) discovered distinct bowling techniques and therefore called for each spin bowling type to have separate coaching models. Some of the distinct differences between the two spin types included a shorter stride length and spin rate for off spin bowlers, whereas a faster approach speed and flexing elbow action during arm acceleration for leg spinners (Beach et al. 2016). Other biomechanical research into spin bowling has discovered that passive range of motions in hips and shoulders can significantly impact performance (Sanders et al. 2019). Further research has been conducted into the kinematic and movement parameters associated with the production of spin (Chin et al. 2009; Mathankar 2020; Sanders et al. 2018). Despite this, there appears to be a lack of investigation into the effectiveness of spin bowling and their numerous variations in real match situations at the elite level. This is somewhat surprising considering that the tactical aspects of fast bowling have been the subject of extensive investigation (Feros et al. 2013; Jamil et al. 2022, 2023; Mehta et al. 2022).

Another important variable overlooked in limited overs cricket research are the bowling–batting match‐ups. That is, the handedness of the bowler (right or left) against the handedness of the batter (right or left). Previous research has revealed that the angle of delivery can impact bowling performances (Justham et al. 2010) and the angles of delivery will depend somewhat on the handedness of the batters and bowlers in direct competition. The importance of the bowler–batter match‐ups requiring investigation is further emphasised when considering the prevalence of left‐handedness in elite cricket is higher than that of the general population, 33% compared to 15.2% (Mann et al. 2016). Furthermore, some studies have discovered that left‐handed batters have higher batting averages, bat for longer periods of time and strike a greater number of four‐run boundaries relative to their right‐handed counterparts (Connor et al. 2020).

This study focuses exclusively on T20 cricket, as some consider this to be the hardest form of white‐ball cricket, due in part to the increased physiological demands for batters and fielders and the reduced margin for error for bowlers (Jamil et al. 2021; Mehta et al. 2022; Scanlan et al. 2016). In addition, this study will focus exclusively on spin bowling due to the reasons mentioned above. The main aim of a spin bowler is to bowl the cricket ball with rapid rotation, designed to make the ball deviate from its normal trajectory upon pitching (bouncing), thus making it more challenging for the batter to hit the ball successfully (Govindasamy et al. 2018). There are two types of traditional spin bowling, off spin and leg spin (Chin et al. 2009) and leg spin is generally considered the more difficult to bowl as a result of minimising the trade‐off between spin rate and accuracy (Woolmer et al. 2008). Common bowling variations within these two spin types include what are referred to as the googly, slider, carrom ball, doosra and many others (Chin et al. 2009; Justham et al. 2010). The present study will examine many of these variations in an attempt to identify the most effective types of variation with regards to the two main bowling KPI's in limited overs cricket of taking wickets and preventing the concession of runs (Mehta et al. 2022).

Although previous research examining the relationship between spin bowling and bowling success are limited relative to fast bowling studies, there have been some interesting findings. For example, spin bowlers were found to neither increase nor decrease the chances of success in the Indian Premier League T20 cricket (Petersen et al. 2008). Najdan et al. (2014) reported a slight disadvantage of bowling spin during the last six overs in T20 cricket, therefore suggested using more defensive spin bowlers during the middle overs (i.e., 11–14) of an innings as they were less likely to concede boundaries. Another study discovered spinners to have a negative impact on team success as a result of their increased economy rate (Irvine and Kennedy 2017). However, it is important to highlight that these aforementioned studies were not specifically researching spin bowling but instead were part of wider analysis into batting and bowling performances. Furthermore, these previous studies did not differentiate between the two types of spin bowling nor consider the handedness of the bowler and/or batter and therefore overlooked the potential impact bowler–batter match‐ups can have on performance. To the best of the authors knowledge, no research has been published that explicitly analyses the effectiveness of spin bowling variations in a match setting at the professional elite level, while also analysing the effectiveness of these variations when controlling for bowler‐batter handedness.

Therefore, the present study will focus on identifying the most effective spin variations in an elite international and franchise T20 cricket whilst also assessing bowler–batter match‐ups. From a practical perspective, the findings could be used to inform on‐field decision‐making with regards to when to use spin bowlers during an innings. Furthermore, by establishing the most effective spin variation, this study could also inform future coaching practice and aid player recruitment in franchise cricket.

## 2 Methods

### 2.1 Design and Data

This study consisted of two analyses. The primary analysis focussed on bowler–batter match‐ups and aimed to determine whether there were any significant relationships between any one of multiple, right‐hand and left‐hand bowler–batter combinations. The secondary analysis aimed to identify the most effective spin bowling variation at the elite level in cricket with regards to the frequently used key performance indicators (KPI's) of ‘wickets taken’ and ‘runs conceded’ as measures of bowling performance (Douglas and Tam 2010; Jamil et al. 2023; Mehta et al. 2022). Full definitions of all cricket terms used in this study are presented in Table 1.

**TABLE 1:** Definitions list for all variables provided by the data supplier.

Variable	Definition
Right arm off spinner	A right‐handed bowler who specialises in bowling off spin.
Left arm orthodox	A left‐handed bowler who specialises in bowling off spin.
Right arm leg spinner	A right‐handed bowler who specialises in bowling leg spin.
Left arm unorthodox	A left‐handed bowler who specialises in bowling leg spin.
Off break	The stock delivery from a right‐arm off spinner or a left‐arm orthodox bowler. For a right‐arm off spinner, the ball will turn from off to leg for a right‐handed batter and from leg to off for a left‐handed batter. For a left‐arm orthodox bowler, the ball will turn from leg to off for a right‐handed batter and from off to leg for a left‐handed batter.
Arm ball	A delivery from a right‐arm off‐spinner or a left‐arm orthodox bowler, which is intended to go straight on off the pitch, often involving drift through the air.
Carrom ball	A delivery from a spinner that is flicked out of the front of the hand.
Leg spinner	The stock delivery from a right‐arm leg‐spinner or left‐arm unorthodox bowler. For a right‐arm leg spin bowler, the ball will turn from leg to off for a right‐handed batter. For a left‐arm unorthodox bowler, the ball will turn from off to leg to a right‐handed batter. (The opposite is true for left‐handed batters).
Googly	The variation from a right‐arm leg‐spinner or left‐arm unorthodox bowler that turns the opposite way to the leg‐spinner. It is usually bowled out of the back of the hand. For a right‐arm leg spin bowler, the ball will turn from off to leg for a right‐handed batter. For a left‐arm unorthodox bowler, the ball will turn from leg to off to a right‐handed batter. (The opposite is true for left‐handed batters).
Quicker ball	A delivery by a spinner that is bowled deliberately quicker than their usual pace.

Spin bowling performance data from several T20 elite tournaments were analysed (ICC World Cup Twenty20 2016, Bangladesh Premier League 2019/20, Caribbean Premier League 2020, Indian Premier League 2020, KFC T20 Big Bash League 2019/20 and the Pakistan Super League 2020). Performance data were obtained from Opta (London, UK), known for their high levels of reliability (Jamil et al. 2023). The original dataset comprised of 23,521 balls bowled; however, some bowling variations were removed from the original dataset due to the number of instances being fewer than 1% of the total number of balls bowled. The variations that were ultimately removed included the doosra (*n* = 51), the flipper (*n* = 158), no movement (*n* = 29), the slider (*n* = 118) and the top spinner (*n* = 81). Consequently, the final sample for each analysis consisted of 23,084 balls bowled. Ethical approval for this study was obtained by the ethics committee of the local institution.

### 2.2 Primary Analysis

**FIGURE 1:**
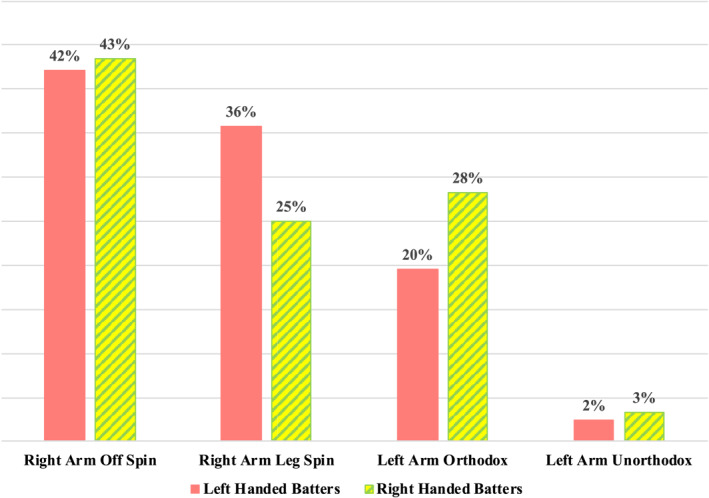
Proportion of balls faced by left‐handed and right‐handed batters from each type of bowler.

In this analysis, the performances of right‐handed and left‐handed batters against spin bowling were assessed in isolation. The bowling KPI's of wickets taken and runs conceded were categorised as ‘yes’ and ‘no’ for each of the balls bowled to right‐handed batters (*n* = 15,056) and balls bowled to left‐handed batters (*n* = 8028). The balls bowled were grouped into one of four categories depending upon the type of bowler and these categories consisted of right‐handed off spinners, right‐handed leg spinners, left‐handed orthodox (off‐spinners) and left‐handed unorthodox (leg‐spinners). The proportions of balls faced by left‐handed and right‐handed batters from each type of bowler are presented in Figure 1.

### 2.3 Secondary Analysis

**FIGURE 2:**
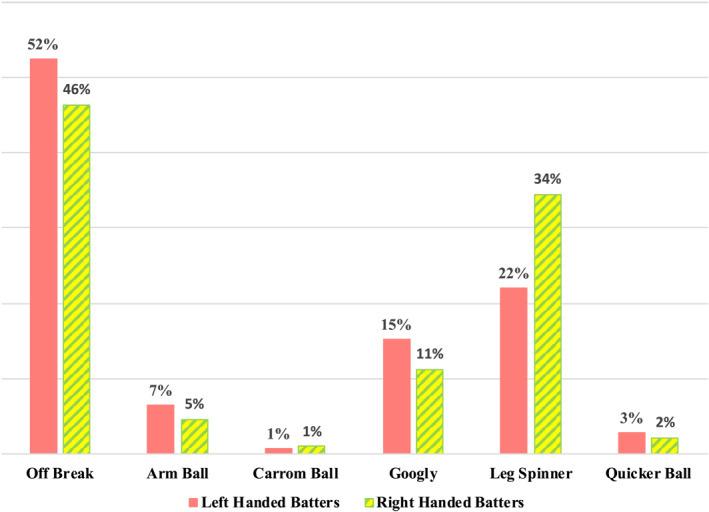
Proportion of balls faced by left‐handed and right‐handed batters from each type of bowling variation.

In this analysis, the performances of right‐handed and left‐handed batters against spin bowling were assessed in isolation. The bowling KPI's of wickets taken and runs conceded were categorised as ‘yes’ and ‘no’ for each of the balls bowled to right‐handed batters (*n* = 15,056) and balls bowled to left‐handed batters (*n* = 8028). The balls bowled were grouped into one of six categories depending upon the type of ball they were and these categories consisted of the arm ball, the carrom ball, the googly, the leg‐spinner, the off‐break and the quicker ball. The proportions of balls faced by left‐handed and right‐handed batters from each type of bowling variation are presented in Figure 2.

### 2.4 Statistical Analysis

**FIGURE 3:**
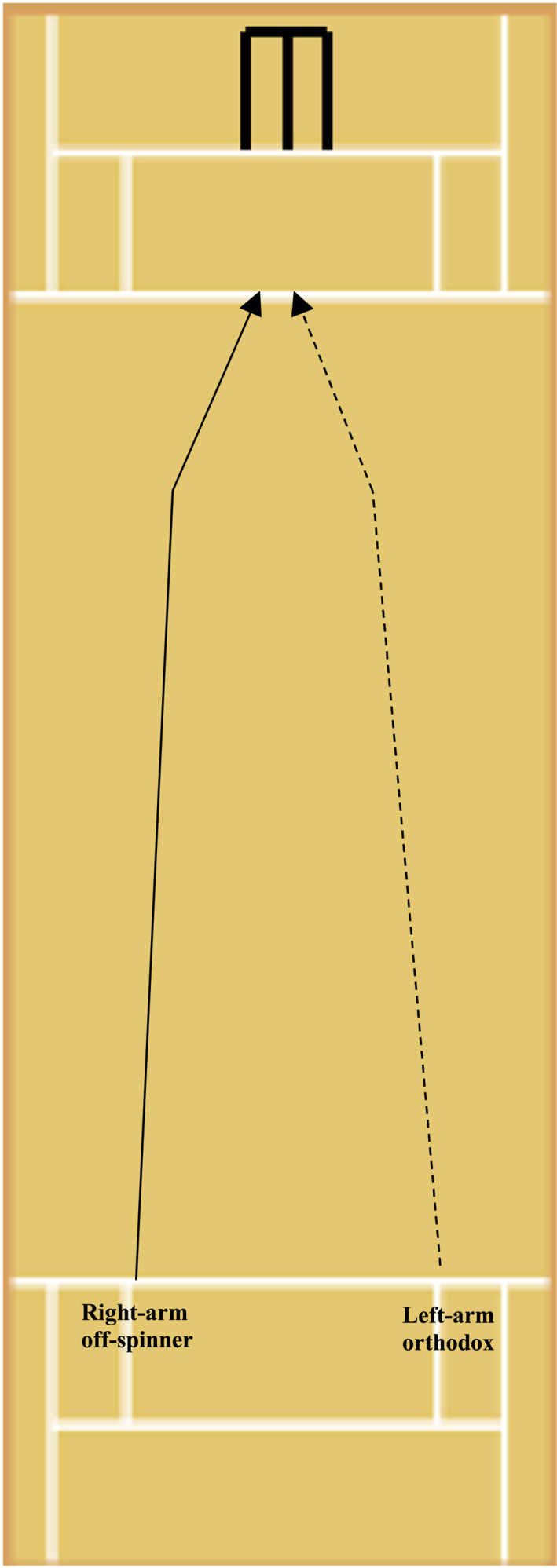
The trajectories of a right‐arm off spinner and left‐arm orthodox bowler bowling their stock delivery (off‐break). Please note that this figure depicts bowling from over the wicket. Bowling from around the wicket would alter the release point of the ball (from the opposite side) but not the spin direction. Landing zones and degrees of spin are approximations.

**FIGURE 4:**
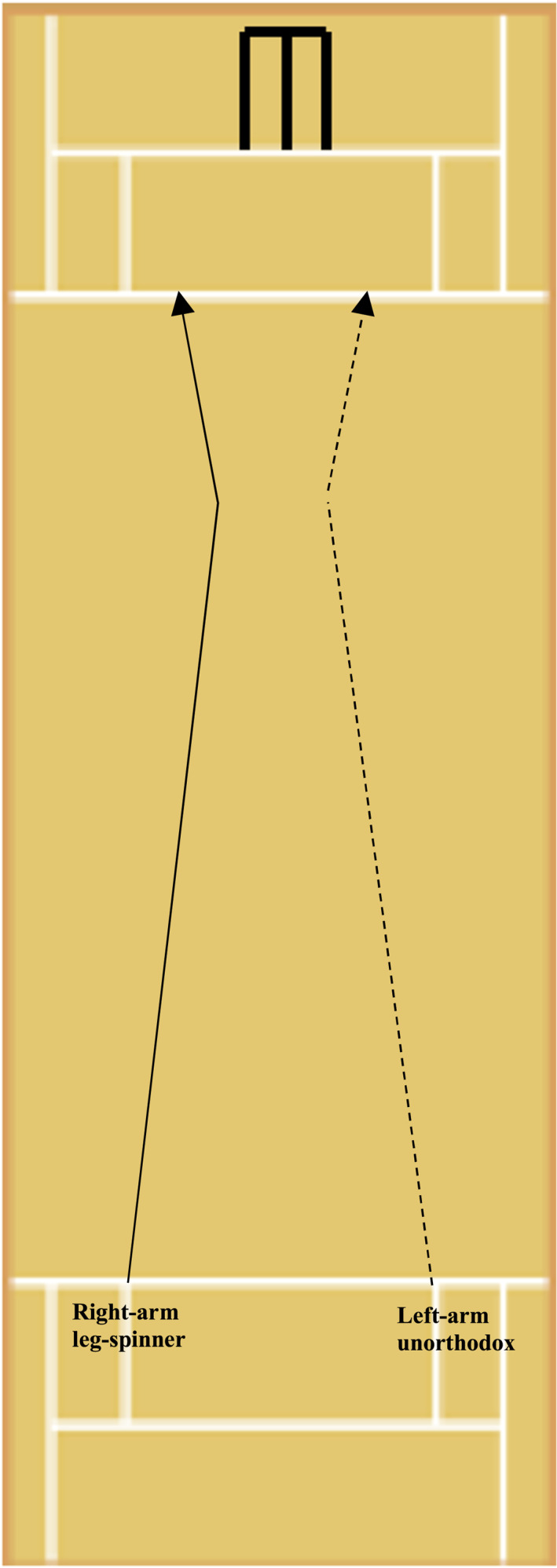
The trajectories of a right‐arm leg‐spinner and left‐arm unorthodox bowler bowling their stock delivery (leg‐spinner). Please note that this figure depicts bowling from over the wicket. Bowling from around the wicket would alter the release point of the ball (from the opposite side) but not the spin direction. Landing zones and degrees of spin are approximations.

In this study, a total of 23,084 balls bowled by professional spin bowlers were analysed. Figures 3 and 4 illustrate the usual ball trajectories of many of the bowling variations defined in Table 1. A series of independent logistic regressions were conducted (8 in total) with runs conceded/wickets taken the dependent binary variables and the categorical independent variables of bowler type and bowling variation, which were modelled to predict the logarithm of the odds of runs being conceded and wickets being taken (Peng et al. 2002). All balls bowled were treated as independent observations due to them having been bowled in non‐homogenous conditions (i.e., at different times, with unique ball conditions and during different match states) and also due to the large degree of variability exhibited ball to ball (i.e., speed of ball bowled, degrees of lateral movement, XY landing zones, field positions and outcome of ball bowled etc). All statistical analyses were performed within the JASP (Amsterdam, Netherlands) software Version 0.14.

## 3 Results

### 3.1 Primary Analysis (Bowler Type)

#### 3.1.1 Bowling to Left‐Handed Batters

The results of the primary analysis for left‐handed batters are presented in Table 2. Bowling right‐arm leg spin to a left‐handed batter will likely result in an increased 30% chance of conceding runs relative to the reference category of right‐arm off spin (*p* < 0.001). Bowling left‐arm orthodox to a left‐handed batter will also likely result in an increased 42% chance of conceding runs relative to the reference category of right‐arm off spin (*p* < 0.001). The type of bowler did not have a significant impact upon taking wickets when bowling to left‐handed batters.

**TABLE 2:** Logistic regression results for left‐handed batters and the type of bowler.

Variable	Runs conceded (0 = No and 1 = yes)					Wickets taken (0 = No and 1 = yes)				
	Estimate	Odds ratio	*p*‐value	BEXP (lower)	BEXP (upper)	Estimate	Odds ratio	*p*‐value	BEXP (lower)	BEXP (upper)
Intercept	0.373	1.452	< 0.001^a^	1.3566	1.5542	−3.086	0.046	< 0.001^a^	0.0389	0.0538
Right‐arm leg spin	0.260	1.298	< 0.001^a^	1.1712	1.4376	0.137	1.147	0.252^b^	0.9076	1.4492
Left‐arm orthodox	0.353	1.424	< 0.001^a^	1.2561	1.6128	0.053	1.054	0.719^b^	0.7914	1.4035
Left‐arm unorthodox	0.256	1.292	0.085^b^	0.9646	1.7315	0.283	1.326	0.360^b^	0.7240	2.4303

*Note:* Ref category = right‐arm off spin.

^a^ = Significant at 95% CI.

^b^ = Nonsignificant.

#### 3.1.2 Bowling to Right‐Handed Batters

The results of the primary analysis for right‐handed batters are presented in Table 3. Bowling left‐arm orthodox (off spin) to a right‐handed batter will likely result in a decreased 8% chance of conceding runs relative to the reference category of right‐arm leg spin (*p* = 0.035). However, bowling left‐arm orthodox (off spin) or right‐arm off spin to a right‐handed batter will likely result in a significantly decreased likelihood of taking a wicket relative to the reference category of right‐arm leg spin by 21% and 20%, respectively (*p* = 0.014 and *p* = 0.025).

**TABLE 3:** Logistic regression results for right‐handed batters and the type of bowler.

Variable	Runs conceded (0 = No and 1 = yes)					Wickets taken (0 = No and 1 = yes)				
	Estimate	Odds ratio	*p*‐value	BEXP (lower)	BEXP (upper)	Estimate	Odds ratio	*p*‐value	BEXP (lower)	BEXP (upper)
Intercept	0.576	1.780	< 0.001^a^	1.6922	1.8701	−2.917	0.054	< 0.001^a^	0.0485	0.0603
Right‐arm off spin	−0.006	0.994	0.883^b^	0.9148	1.0790	−0.219	0.803	0.025^a^	0.6623	0.9734
Left‐arm orthodox	−0.085	0.918	0.035^a^	0.8487	0.9940	−0.233	0.792	0.014^a^	0.6584	0.9531
Left‐arm unorthodox	0.188	1.207	0.056^b^	0.9950	1.4637	−0.091	0.913	0.673^b^	0.5975	0.8336

*Note:* Ref category = right‐arm leg spin.

^a^ = Significant at 95% CI.

^b^ = Nonsignificant.

### 3.2 Secondary Analysis (Bowling Variations)

#### 3.2.1 Bowling to Left‐Handed Batters

The results of the secondary analysis for left‐handed batters are presented in Table 4. Bowling the arm ball variation is significantly more likely to result in runs being conceded by 27% relative to the reference category of off spinners (*p* = 0.015). Bowling the leg spinner variation is also significantly more likely to result in runs being conceded by 41% relative to the reference category of off spinners (*p* < 0.001). The carrom ball, quicker ball and googly variations revealed no significant effects on runs conceded when bowled to left‐handed batters. No bowling variations significantly impacted the chances of taking wickets when bowling to left‐handed batters.

**TABLE 4:** Logistic regression results for left‐handed batters and the bowling variation.

Variable	Runs conceded (0 = No and 1 = yes)					Wickets taken (0 = No and 1 = yes)				
	Estimate	Odds ratio	*p*‐value	BEXP (lower)	BEXP (upper)	Estimate	Odds ratio	*p*‐value	BEXP (lower)	BEXP (upper)
Intercept	0.455	1.577	< 0.001^a^	1.4814	1.6770	−3.035	0.048	< 0.001^a^	0.0447	0.0555
Arm ball	0.238	1.268	0.015^a^	1.0471	1.5357	−0.309	0.734	0.218^b^	0.4489	1.2009
Carrom ball	−0.168	0.846	0.513^b^	0.5112	1.3979	0.039	1.040	0.947^b^	0.3234	3.3468
Googly	−0.031	0.970	0.642^b^	0.8513	1.1041	0.263	1.300	0.065^b^	0.9841	1.7177
Leg spinner	0.342	1.408	< 0.001^a^	1.2511	1.5841	−0.099	0.906	0.478^b^	0.6887	1.1912
Quicker ball	0.104	1.110	0.458^b^	0.8428	1.4608	0.039	1.040	0.901^b^	0.5582	1.9387

*Note:* Ref category = off break.

^a^ = Significant at 95% CI.

^b^ = Nonsignificant.

#### 3.2.2 Bowling to Right‐Handed Batters

The results of the secondary analysis for right‐handed batters are presented in Table 5. Bowling the leg spinner variation is significantly more likely to result in runs being conceded by around 16% relative to the reference category of off spinners (*p* = 0.05). Bowling the carrom ball variation is significantly less likely to result in runs being conceded by around 27% relative to the reference category of off spinners (*p* < 0.001). Bowling the googly variation is also significantly less likely to result in runs being conceded by around 17% relative to the reference category of off spinners (*p* < 0.001). The quicker ball and arm ball variations revealed no significant effects on runs conceded when bowled to right‐handed batters. Bowling the carrom ball variation was also revealed to be significantly more likely to result in wickets being taken by a factor of 2:1 (twice as likely) relative to the reference category of off spinners (*p* = 0.016). Similarly, bowling the googly variation was also revealed to be significantly more likely to result in wickets being taken by around 72% relative to the reference category of off spinners (*p* < 0.001).

**TABLE 5:** Logistic regression results for right‐handed batters and the bowling variation.

Variable	Runs conceded (0 = No and 1 = yes)					Wickets taken (0 = No and 1 = yes)				
	Estimate	Odds ratio	*p*‐value	BEXP (lower)	BEXP (upper)	Estimate	Odds ratio	*p*‐value	BEXP (lower)	BEXP (upper)
Intercept	0.532	1.703	< 0.001^a^	1.6226	1.7878	−3.164	0.042	< 0.001^a^	0.0375	0.0476
Arm ball	−0.010	0.990	0.906^b^	0.8428	1.1642	−0.081	0.922	0.698^b^	0.6126	1.3882
Carrom ball	−0.302	0.732	0.050^a^	0.5363	0.9999	0.711	2.037	0.016^a^	1.1411	3.6328
Googly	−0.193	0.825	< 0.001^a^	0.7401	0.9194	0.543	1.722	< 0.001^a^	1.3771	2.1533
Leg spinner	0.144	1.155	< 0.001^a^	1.0704	1.2448	0.088	1.092	0.333^b^	0.9139	1.3047
Quicker ball	0.121	1.129	0.303^b^	0.8967	1.4219	0.292	1.339	0.242^b^	0.8204	2.1858

*Note:* Ref category = off break.

^a^ Significant at 95% CI.

^b^ Nonsignificant.

## 4 Discussion

This study had two main aims. Firstly, this study examined whether bowler–batter match‐ups are an important consideration at the international and franchise level in T20 cricket. The results indicate that bowler–batter match‐ups do matter when bowling to both right‐handed and left‐handed batters. Specifically, with regards to conceding runs, opposing match‐ups where the ball naturally spins away from the batters appears to be an effective strategy (in other words, bowling right‐arm off spin bowlers to left‐handed batter or left‐arm orthodox bowlers to right‐handed batters). However, this strategy of opposing match‐ups is less likely to take wickets, at least when bowled to right‐handed batters, as this study revealed right‐handed leg spinners to be more effective at getting right‐handed batters out. Ultimately, bowling–batting match‐ups need to be taken into consideration at the elite T20 level and the results obtained in this study could potentially inform on‐field decision‐making.

Secondly, this study examined which specific spin variations were most effective with regards to helping spin bowlers fulfil their main bowling objectives of preventing the concession of runs and taking wickets. Results revealed that when bowling to left‐handed batters, the off‐spinner variation was the most effective at restricting runs conceded with the arm ball and leg spinner both more likely to concede runs. However, when bowling to right‐handed batters, the ‘carrom ball’ and ‘googly’ variations were both revealed to be particularly effective at restricting runs conceded as well as taking wickets, suggesting that they are both particularly effective bowling variations for spin bowlers to possess. Furthermore, similarly to the results obtained when bowling to left‐handed batters, the traditional leg‐spinning stock delivery was revealed to be more likely to concede runs when bowling it to right‐handed batters.

The results of the primary analysis revealed that right‐arm off spinners can restrict the runs scored by left‐handed batters. Similarly, left‐arm orthodox bowlers can restrict the runs scored by right‐handed batters. When interpreted in unison, these results suggest that opposing match‐ups where the ball naturally spins away from the batter is an effective method of restricting the runs scored by both right‐handed and left‐handed batters. These results could be partially explained by the natural angle of deliveries moving across and away from the batters. Justham et al. (2010) reported that spin bowlers bowled the vast majority of their deliveries (71%) from over the wicket to left‐handed batters, implying that bowlers prefer to bowl on a trajectory where the ball moves across the batter. In addition, the majority of deliveries to left hand batters from over the wicket are the bowlers stock delivery. Furthermore, Noorbhai and Noakes (2019) reported that batting stroke‐play becomes easier when a batter's wrists are kept close to their own body as the level of muscular effort required to play a shot is reduced. This could be a reason why modern‐day spin bowlers favour spinning the ball away from the batter as they would inevitably force the batters to play away from their body.

The results of the primary analysis also revealed that right‐arm leg spinners were more likely to take the wickets of right‐handed batters. Previous research has revealed that being able to angle the ball in and then spin the ball away from the batter (as would naturally occur when right arm leg spinners bowl to right‐handed batters) can lead to reduced shot control from batters (Spratford et al. 2020). It is for this reason that cricket coaches have previously claimed that possessing a bowler capable of spinning the ball away from the batter, while also bowling with good control could be a potential match winner (Woolmer et al. 2008). However, Woolmer et al. (2008) did caution that bowling this type of spin without control can have undesired effects on performance, particularly as this would present batters with increased scoring opportunities. Interestingly, our analysis only revealed this trend for right‐handed bowler–batter match‐ups and not when left‐arm unorthodox bowlers bowled to left‐handed batters. This could be partially due to the ‘*negative frequency*’ effect, whereby bowlers train predominantly against right‐handed batters and are therefore more accustomed to their behaviours (Connor et al. 2020) as opposed to their left‐handed counterparts. The rarity of this particular match‐up between left‐arm unorthodox bowlers and left‐handed batters is also highlighted in the dataset analysed in this study, as only 2% of all events analysed across all 6 tournaments comprised of this particular match‐up.

The results of the secondary analysis revealed that the traditional ‘leg‐spinner’ resulted in significantly increased likelihood of conceding runs when bowling it to both right‐handed and left‐handed batters. This result could be partially explained by batters being able to identify the leg‐spinner through advanced cue information in the bowlers action. Renshaw and Fairweather (2000) reported that professional and amateur batters found the leg‐spinning delivery the easiest to anticipate when asked to discriminate between the different delivery variations from a spin bowler. In addition, leg‐spin bowlers bowling with a lack of control could also have made it easier for batters to score runs. Previous studies have revealed that mastering the art of leg spin is incredibly difficult relative to off‐spin (Beach et al. 2016) and that the increased spin rate of leg spin often comes at the cost of reduced accuracy, which also explains the relatively lower proportion of leg‐spinners in elite cricket (Beach et al. 2016; Woolmer et al. 2008). The arm ball variation was also revealed to be significantly more likely to result in conceding runs when bowled to left‐handed batters. Some may consider this a somewhat surprising result, as previous research has revealed that balls that exhibit some form of lateral movement as they approach the batter is more effective at enabling bowlers to fulfil their objectives (Mehta et al. 2022). However, this study by Mehta et al. (2022), focused exclusively on fast bowlers. Spin bowlers generally bowl at a much slower pace (MacDonald Wells et al. 2018), which could potentially offer batters more time to anticipate the drift (during a successfully executed arm ball) and thus react (Müller and Abernethy 2006). This particular result could also be partially explained by the ‘*negative frequency*’ effect mentioned above, as bowlers would more likely practice the arm‐ball variation against right‐handed batters in training environments (Connor et al. 2020); however, this particular finding requires further investigation.

The results of the secondary analysis also revealed that the carrom ball and googly variations are particularly effective at both restricting runs and taking wickets, but only when they are bowled to right‐handed batters. Previous studies have suggested that at the elite level ‘mystery’ spin bowlers capable of bowling multiple variations of deliveries with a high degree of control should be more frequently used as strike (wicket taking) bowlers during the middle phases of T20 innings due to their ability to deceive opposing batters (Irvine and Kennedy 2017; Jamil et al. 2023). The fact that this trend was only discovered for right‐handed batters as opposed to their left‐handed counterparts again could be at least partially due to the ‘*negative frequency*’ effect detailed above; however, this warrants further research.

The results of this study have confirmed significant relationships between bowler–batter match‐ups with regards to bowler types and specific spin variations in the elite T20 format of cricket. Specifically, the results of this study have revealed that the strategy of opposing match‐ups where the ball naturally spins away from the batters could restrict runs scored for both right‐handed and left‐handed batters. Furthermore, right arm leg‐spin bowlers are revealed to be more likely to get right‐handed batters out. In addition, the results of this study have also highlighted the value of T20 teams possessing an elite wrist spin bowler capable of bowling the ‘googly’ variation as this has been revealed to be particularly effective at taking wickets as well as restricting runs scored for right‐handed batters. The ‘carrom ball’ variation is also revealed to be particularly effective at taking wickets as well as restricting runs scored for right‐handed batters. From a practical perspective, the results of this study can inform future coaching practice. Additionally, the findings of this study could inform the tactics and strategies of the on‐field captains during a match as well as team selection decisions from the captain/head coach and opposition scouts for upcoming fixtures. Furthermore, the results of this study could potentially encourage the selection of multiple wrist spinners within the same team, which appears to be somewhat of a rarity in elite cricket and also potentially influence recruitment decisions made in elite franchise T20 cricket leagues.

This study was not without its limitations. Firstly, only the T20 format was investigated. It could well be the case that the bowler–batter match‐up trends discovered in this study as well as the spin variation results are not homogenous and do not pertain to other forms of cricket such as one‐day, test and the newly conceived ‘The Hundred’. Furthermore, data on other variables that could have potentially affected the results, such as bowling angles (over and around the wicket), batter foot movement and pitch characteristics (slow or fast pitch), were absent in this study. Finally, only spin bowling was considered in this research. Future studies could expand on this research and incorporate the variables listed above as well as investigate trends in the different formats of elite cricket.

## 5 Conclusion

In conclusion, this study suggests that bowler–batter match‐ups do matter at the elite T20 level. Specifically, opposing match‐ups where the ball naturally spins away from batters is revealed to be an effective strategy at restricting runs conceded when bowling to both right‐handed and left‐handed batters. Furthermore, right arm leg spinners are revealed to be effective at taking the wickets of right‐handed batters. With regards to spin variations, the ‘googly’ and ‘carrom ball’ were both revealed in this study to be particularly effective at both taking wickets and restricting runs scored when bowling it to right‐handed batters in the T20 format. The traditional ‘leg‐spinning’ delivery was revealed to result in an increased likelihood of conceding runs when bowling it to both right‐handed and left‐handed batters, implying controlled leg spin bowling is crucial in T20 cricket. From a practical perspective, the findings of this study could inform on‐field decision making of team captains, playing strategies and team selection devised by captains and head coaches as well as the recruitment policies of franchise cricket teams and general coaching practice.

## Ethics Statement

This study was approved by the University of Suffolk Research Ethics Committee (RDU21/012).

## Conflicts of Interest

The authors declare no conflicts of interest.
